# Effectiveness of a Community-based Group Mindfulness Program tailored for Arabic and Bangla-speaking Migrants

**DOI:** 10.1186/s13033-021-00456-0

**Published:** 2021-04-13

**Authors:** Ilse Blignault, Hend Saab, Lisa Woodland, Haider Mannan, Arshdeep Kaur

**Affiliations:** 1 Translational Health Research Institute, Western Sydney University, Locked Bag 1797, Penrith, NSW 2751 Australia; 2 Multicultural Health Service, South Eastern Sydney Local Health District, PO Box 1614, Sydney, NSW 2001 Australia; 3 Priority Populations, Population and Community Health, South Eastern Sydney Local Health District, PO Box 1614, Sydney, NSW 2001 Australia

**Keywords:** Mindfulness-based intervention, Evaluation, Arabic language, Bangla language, Cultural adaptation, Cultural competence, Migrant, Australia

## Abstract

**Background:**

Migrant communities are often underserved by mainstream mental health services resulting in high rates of untreated psychological distress. This collaborative study built on evidence that mindfulness-based interventions delivered in-language and culturally tailored were acceptable and clinically effective for Arabic speakers in Australia. It aimed to establish whether a group mindfulness program produced expected outcomes under normal operational conditions, and to test its scalability and its transferability to Bangla speakers.

**Methods:**

A 5-week mindfulness program was delivered to 15 Arabic-speaking and 8 Bangla-speaking groups in community settings. The mixed-methods evaluation incorporated a pre-post study. Descriptive statistics were used to summarise the socio-demographic data, group attendance and home practice. Differences in DASS 21 and K10 scores from pre to post-intervention were tested using the nonparametric sign test for paired samples (two-sided). Multiple linear regression analysis was performed to determine the effects of selected sociodemographic variables, group attendance and home practice on clinical outcomes, based on intention to treat. Content analysis was used to examine the qualitative data.

**Results:**

The program attracted 168 Arabic speakers and 103 Bangla speakers aged 16 years and over, mostly women. Cultural acceptability was evident in the overall 80% completion rate, with 78% of Arabic speakers and 84% of Bangla speakers retained. Both language groups showed clinically and statistically significant improvements in mental health outcomes on the DASS21 and K10. Thirty new referrals were made to mental health services. Participant feedback emphasised the benefits for their everyday lives. All but one participant reported sharing the mindfulness skills with others.

**Conclusions:**

Across multiple and diverse groups of Arabic and Bangla speakers in Sydney, the community-based group mindfulness program was shown to have high levels of cultural acceptability and relevance. It resulted in clinically and statistically significant improvements in mental health outcomes, facilitated access to mental health care and boosted mental health literacy. This innovative, low-intensity, in-language mental health intervention that was originally developed for Arabic speakers is scalable. It is also transferable—with cultural tailoring—to Bangla speakers.

**Supplementary Information:**

The online version contains supplementary material available at 10.1186/s13033-021-00456-0.

## Background

Australia today is home to people who come from over 190 countries and speak more than 300 languages; it is a strong and successful multicultural society [1]. Arabic speakers constitute a relatively large and long-established non-English-speaking language group. In recent decades they have been joined by arrivals from a range of countries in the Middle East and North Africa, as well as new and emerging communities from Asia [2, 3]. Despite high levels of need, migrants and refugees face multiple barriers in accessing mental health care [4]. Those from non-English-speaking countries are often underserved by mainstream mental health services, particularly when translation is required [5].

In New South Wales (NSW), Australia’s most populous state, 28% of residents were born overseas, 47% have at least one parent who was born overseas and 25% speak a language other than English at home [6]. Arabic is the fourth most common language other than English spoken in South Eastern Sydney Local Health District (SESLHD) [5] and the second most common in Sydney Local Health District (SLHD) [7]. Speakers of the Bangla (or Bengali) language constitute a significant new and emerging community in both health districts [8].

This paper reports on the third phase of an ongoing program of research into translated and culturally adapted mindfulness-based interventions, conducted in 2017–19. Earlier studies with Arabic-speaking community members (Phase 1 in 2014–15 [9] and Phase 2 in 2016–17 [10, 11]) demonstrated that mindfulness was compatible with their cultural and religious practices and improved psychological wellbeing. Those studies showed that an Arabic Mindfulness CD was effective when used as a self-management tool in the home setting [9] and within a group program [10, 11]. Qualitative data collected in Phase 2 indicated that the mindfulness program increased participants’ understanding of the connection between physical and emotional pain, their ability to deal with past trauma and everyday problems, and enabled more focused religious practice. The group setting provided opportunities for connecting with other women and peer support [10, 11].

Based on these promising results, the Central and Eastern Sydney Primary Health Network (CESPHN), whose boundaries align with SESLHD and SLHD, commissioned the SESLHD Multicultural Health Service to deliver the group mindfulness program to Arabic- and Bangla-speaking communities in their region, and evaluate process and outcomes (Phase 3). This collaborative multi-site project brought together a large primary care organisation, two mental health services and many community groups. The broader project objectives included delivering the program to the target communities in order to reduce psychological distress, depression, anxiety and stress; developing in-language resources to support the program; and training bilingual mental health clinicians and community workers in mindfulness-based interventions. As in the earlier studies, the main evaluation questions concerned the acceptability and clinical utility of the group mindfulness program for each community. We also examined the combined dataset to determine the effects of selected sociodemographic variables and intervention exposure on mental health outcomes.

In terms of the Sax Institute’s Translational Research Framework [12], Phase 3 was positioned primarily as an effectiveness study in that it aimed to establish whether the group mindfulness program produced the expected outcomes under normal operational conditions. In addition, the project tested its transferability to another language group and its scalability, i.e. integration of this innovative program into the wider health system.

## Methods

### Target group and setting

The group mindfulness program, which targeted Arabic- and Bangla-speaking community members aged 16–65 years, was widely advertised within health and community networks through flyers, social media and word of mouth. It was delivered in suburbs with high numbers of Arabic-speaking or Bangla-speaking residents at venues regularly used by the target population, such as migrant resource centres and community facilities attached to mosques and churches. Most groups were held in the morning; a couple in the evening. Refreshments were provided for all groups. Child minding was provided when required.

### Intervention

Box 1 provides an overview of the program content. Development of the Arabic Mindfulness CD, an adaptation of a popular English-language resource, is described in Blignault et al. [9]. A new Bangla Mindfulness CD was developed as part of Phase 3. Other program materials included a 43-page participant handbook and handouts that were translated by accredited translators into Arabic and Bangla. A Bangla-speaking working group of bilingual mental health professionals and community members provided advice on translation and cultural adaptation of the program for their community.

#### Box 1. Overview of the CALD Group Mindfulness Program

**Table:** 

*Group session 1: Introduction to Mindfulness* Aim: To present mindfulness concepts to participants, highlight its origins and benefits, and explore the cultural and spiritual relevance of mindfulness practices. Mindfulness practice: Grounding exercise with sensory awareness; Mindfulness breathing. *Group session 2: Stress and Stress Management* Aim: To educate participants about stress and the stress response from an evolutionary perspective, help them identify their own stress symptoms, and provide them with mindfulness skills to manage stress. Mindfulness practice: Grounding exercise with sensory awareness; Mindfulness breathing; Leaves on a Stream. *Group session 3: Self and the Observing Self* Aim: To identify judgement and self-judgement and teach participants to let go and disentangle from negative self-judgement using mindfulness, and guide participants towards establishing or recognising their own sense of self. Mindfulness practice: Grounding exercise with mindfulness breathing; Body scan with Leaves on a Stream. *Group session 4: Loving Kindness and Self-Compassion* Aim: To guide participants towards reinforcing their sense of self and nurturing that sense of self through loving kindness and self-compassion. Mindfulness practice: Mindfulness breathing with body scan; Loving kindness and self-compassion exercise. *Group session 5: Managing Painful Emotions* Aim: To educate participants about biological and psychological pain and introduce mindfulness practices as a means to reduce suffering. Mindfulness practice: Grounding exercise with sensory awareness; Body scan and sitting with difficult emotions; Mindfulness breathing; Leaves on a Stream	

Participants were required to attend group sessions once a week for five weeks, with home practice in between. Sessions were conducted in Arabic, Bangla, or the group language and English. They were co-facilitated by bilingual psychologists and trained community workers who emphasised the cultural and spiritual relevance of mindfulness practice in their explanations and examples, tailoring the content according to the needs of each group (e.g. young mothers, older women or men). The co-facilitators maintained weekly contact with all participants, providing telephone support between sessions as required. All groups were overseen by the clinical lead (second author, HS) who provided ongoing supervision for the co-facilitators and coordinated referrals for additional mental health care.

Participants who indicated a high level of psychological distress at the start or during the program were followed up by the bilingual psychologist and referred for further support to mental health services, psychologists or the NSW Service for the Treatment and Rehabilitation of Torture and Trauma Survivors (STARTTS).

### Evaluation

#### Design

The mixed-methods evaluation incorporated a pre-post study. Quantitative data were collected at the first and final group sessions. Participants completed a feedback form with open-ended questions at the end of each weekly session. The co-facilitators kept a record of group attendance and referrals and made notes on group processes and participants’ comments during the sessions and afterwards.

#### Measures

The data collection tools comprised a questionnaire capturing sociodemographic data and knowledge and perception of mindfulness, and two standardised psychometric instruments—the K10 + and DASS21. Both instruments are widely used in the Australian context, where the K10 + forms part of the National Outcomes and Casemix Collection [13]. The K10 yields a global measure of psychological distress based on symptoms that a person has experienced in the past month [14]. The K10 + includes four additional questions enquiring about the individual's ability to work and carry out day-to-day activities, the number of times the person has seen a doctor or other health professional due to mental health issues, and how often physical health problems have been the main cause of these issues [15]. The NSW Transcultural Mental Health Centre has produced translated versions in over 27 languages, including Arabic and Bangla [16]. The DASS21 is a 21-item questionnaire that includes three self-report scales measuring depression, anxiety and stress [17, 18]. It has been translated and validated in both Arabic and Bangla [19, 20]. Questions in the weekly feedback form asked which components of the group session were most helpful and not helpful, whether participants had practised the mindfulness exercises at home and, if so, for comments about their experience. The post-program questionnaire included an extra question about sharing the mindfulness skills with others. All evaluation tools were translated into Arabic and Bangla by accredited translators and checked by other accredited translators and community members.

#### Analysis

Quantitative analyses were conducted using IBM SPSS Statistics V26 and SAS 9.4. Descriptive statistics were used to summarise the socio-demographic data, group attendance and home practice. Pre and post-measures of mental health on the K10 + and the DASS21 subscales were compared using the paired-sample sign test (two-sided), as the data were not normally distributed. The null hypothesis for the sign test is the median difference is zero. Multiple linear regression analysis was performed to determine the effects of age, years in Australia, education, language spoken at home, gender, group language, home practice and group attendance on differences between post and pre-measures of mental health. The analysis was conducted with the main effects of the predictors only and with the main effects plus the home practice x group attendance interaction or joint effect. Intention to treat analysis involved multiple imputation all missing data; it was performed 25 times. Multiple linear regression analysis was performed using each imputed data set and the results combined to obtain pooled estimates of regression coefficients and their 95% confidence intervals. Higher scores on both psychometric instruments indicate higher distress, thus a negative post–pre difference indicates improvement in mental health.

Participant feedback in Arabic and Bangla was translated into English by the bilingual co-facilitators for examination. Qualitative content analysis of the data was undertaken manually by two researchers who worked collaboratively [21].

#### Ethics

Ethics approval was obtained from the SESLHD Human Research Ethics Committee. Written consent was obtained by the co-facilitators who went through the Participant Information Sheet (available in Arabic/Bangla and English) and answered any questions. The study was monitored by a steering committee comprising the principal investigators (authors LW, HS and IB) and staff and managers from the health services involved.

## Results

The evaluation findings are organised under five main headings: (1) Participant characteristics, (2) Program adherence, (3) Mental health outcomes, (4) Referrals and (5) Participant experiences.

### Participant characteristics

Over a two-year period, 23 group mindfulness programs were facilitated across the region: 15 for Arabic speakers and 8 for Bangla speakers. Fifteen groups were women only, 1 was men only (Arabic) and 7 were mixed. They attracted a total of 271 participants aged 16 years and over, of whom 218 (80%) completed the program: 131 (78%) Arabic speakers and 87 (84%) Bangla speakers. The group size ranged from 8 to 18 with a mean, median and mode of 13. Those who dropped out mostly did so after one or two sessions. Common reasons included overseas travel, family sickness and the time conflicting with English classes. Using logistic regression, we found there were no differences between dropouts and participants retained on baseline DASS21 and K10 + scores (results not given).

#### Arabic-speaking groups

Across all the Arabic-speaking groups168 participants were recruited; 83% female. Most fell into the 26–35 and 56–65 age groups. Seventy-nine per cent were overseas-born, mostly in Lebanon, Egypt or Iraq; just over half had lived in Australia for eleven or more years. Overall, 42% spoke only Arabic at home and 48% Arabic and English. Sixty-two per cent were Muslims and 54% possessed a post-school qualification. (See Additional file 1 for sociodemographic characteristics of participants recruited).

Pre-intervention, 72% of Arabic speakers recruited reported some degree of psychological distress, with 78% of participants retained scoring ‘mild’ (20–24), ‘moderate’ (25–29), or ‘severe’ (30–50) on the K10. (See Additional file 2 for pre and post K10 and DASS21 categories for participants retained.) Most had little knowledge of mindfulness. (See Additional file 3 for pre and post knowledge and attitudes for participants retained.)

#### Bangla-speaking groups

Across all the Bangla-speaking groups 103 participants were recruited, of whom all but six were female. Most fell into the 26–35 and 36–45 age groups. All but two were born in Bangladesh; 26% had lived in Australia for under three years and 45% for under seven years. Ninety-two per cent were Muslim and most had post-school education including 59% with a university qualification (Additional file 1).

Pre-intervention, 71% of Bangla speakers recruited reported some degree of psychological distress, with 68% of participants retained scoring ‘mild’, ‘moderate’ or ‘severe’ on the K10 (Additional file 2). Most knew little about mindfulness (Additional file 3).

### Program adherence

#### Arabic-speaking groups

Of the 131 participants retained (78% retention rate), 98% attended at least three group sessions and 88% reported at least three weeks of home practice (Table 1). Women were more likely to complete the program than men (84% vs 70%).

**Table 1.** Group attendance and home practice by language group—Participants retained

Number of group sessions attended			Number of weeks home practice		
**Sessions**	**n**	%	**Weeks**	**n**	%
Arabic speakers (N = 131)					
1	0	0	0	3	2.3
2	3	2.3	1	13	9.9
3	13	9.9	2	23	17.6
4	50	38.2	3	47	35.9
5	65	49.6	4	45	34.4
Bangla speakers (N = 87)					
1	0	0	0	0	0
2	0	0	1	6	6.9
3	11	12.6	2	18	20.7
4	30	34.5	3	40	46.0
5	46	52.9	4	23	26.4

#### Bangla-speaking groups

All of the 87 participants retained (84% retention rate) attended at least three group sessions and 93% reported at least three weeks of home practice (Table 1). There were no notable differences between participants who dropped out and those who completed the program**.**

### Mental health outcomes

#### Pre-post comparisons

For both Arabic and Bangla speakers, post-program measures on the DASS21 subscales and the K10 + showed improvement. The median differences in depression, anxiety and stress were statistically significant (all p < 0.001) (Table 2). Clinically, more participants scored ‘normal’ or ‘mild’ and fewer participants scored ‘moderate’ to ‘extremely severe’ (Additional file 2). Similarly, the median differences in psychological distress were statistically significant (all p < 0.001) (Table 2), with more participants scoring ‘well’ to ‘mild’ and fewer participants scored ‘moderate’ to ‘severe’ (Additional file 2). For both language groups, statistically significant differences were observed in days of inability to work and, beyond that, days of cutting down work due to mental health issues in the past four weeks (both p < 0.001) (Table 2).

**Table 2.** Pre-post comparisons on DASS21 and K10 + by language group—Participants retained

Measure	Mean score		Sign test for pre-post change	
Variable	Pre-program	Post-program	**z**	2-sided p
	M (SD)	M (SD)		
Arabic-speakers (N = 131)				
DASS21 subscales				
Depression	9.2 (5.5)	4.2 (4.2)	− 8.48	< 0.001
Anxiety	8.2 (5.5)	3.7 (4.1)	− 7.64	< 0.001
Stress	11.3 (5.2)	5.3 (4.1)	− 9.28	< 0.001
K10 +				
K10 score	27 (9.6)	18.5 (7.1)	− 8.94	< 0.001
Q11. Days of inability to work due to mental health issues in past four weeks	3.9 (5.3)	1.8 (4.4)	− 6.52	< 0.001
Q12. Days of cutting down work due to mental health issues in past four weeks (apart from days in Q11)	9.2 (8.9)	4.1 (6.3)	− 6.19	< 0.001
Bangla-speakers (N = 87)				
DASS21 subscales				
Depression	8.0 (5.3)	4.6 (4.0)	− 5.7	< 0.001
Anxiety	7.2 (5.9)	4.0 (4.1)	− 6.32	< 0.001
Stress	9.4 (5.2)	5.6 (4.1)	− 6.96	< 0.001
K10 +				
K10 score	23.8 (8.1)	17.2 (5.9)	− 7.11	< 0.001
Q11. Days of inability to work due to mental health issues in past four weeks	2.4 (3.6)	0.7 (1.9)	− 5.05	< 0.001
Q12. Days of cutting down work due to mental health issues in past four weeks (apart from days in Q11)	4.8 (6.1)	2.0 (3.7)	− 5.48	< 0.001

#### Regression analyses

Table 3 shows the main effects of age, years in Australia, education, language spoken at home, gender, group language, home practice and group attendance on mental health outcomes (differences between post and pre measures), with 95% confidence intervals. On the K10 + , there was significantly greater reduction in psychological distress only for participants aged 56–65 compared to those aged below 26 (p < 0.01). Also for this age group, there was significantly greater reduction in days of inability to work due to mental health issues compared to those aged below 26 (p < 0.05). On the DASS21, there was a decrease in depression, anxiety and stress with increased age, the effect being significant only for participants aged 46–55 and 56–65 compared to those aged below 26 when the outcome is stress (p < 0.05). There was a significant decrease in stress for participants who had lived in Australia for 11 years or more compared to under four years (p < 0.05). Speaking Bangla rather than Arabic was significantly associated with an increase in post-program scores for depression (p < 0.05), anxiety (p < 0.05) and stress (p < 0.01).

**Table 3:** Multiple linear regression of the effects (and their 95% confidence intervals) of age, years in Australia, education, language spoken at home, gender, group language, home practice and group attendance on mental health outcomes (post–pre differences)

Outcome	Difference in K10 score	Difference in K10 Q11 (Days of inability to work)	Difference in K10 Q12 (Days of cutting down work apart from days in Q11)	Difference in DASS depression	Difference in DASS anxiety	Difference in DASS stress
Covariates	Coefficient (95% CI)	Coefficient (95% CI)	Coefficient (95% CI)	Coefficient (95% CI)	Coefficient(95% CI)	Coefficient (95% CI)
Age group						
(< 26 years)						
26–35	− 1.21 (− 5.23, 2.81)	− 1.54 (− 4.03, 0.96)	− 2.60 (− 6.84, 1.99)	− 0.35 (− 2.71, 2.09)	− 0.93 (− 3.43, 1.58)	− 1.13 (− 2.98, 0.98)
36–45	− 2.29 (− 6.89, 2.31)	− 2.03 (− 4.76, 0.70)	− 2.59 (− 7.18, 1.99)	− 0.75 (− 3.28, 2.01)	− 2.40 (− 5.04, 0.24)	− 1.59 (− 4.02, 0.16)
46–55	− 2.36 (− 7.10, 2.38)	− 2.29 (− 4.98, 0.40)	− 3.81(− 9.03, 1.40)	− 1.69 (− 3.28, 2.01)	− 2.94 (− 6.02,0.14)	− 2.53^c^ (− 4.85, − 0.44)
56–65	− 6.13^b^ (− 10.79, − 1.47)	− 3.03^c^ (− 5.69, − 0.37)	− 1.81 (− 6.30, 2.67)	− 1.44 (− 3.99, 1.28)	− 2.59 (− 5.57, 0.39)	− 3.13^c^ (− 5.33, − 1.24)
Years in Australia						
(< 4)						
4–6	− 1.28 (− 5.79, 3.23)	− 0.70 (− 3.23, 1.83)	− 0.66 (− 5.05, 3.72)	0.49 (− 2.03, 3.24)	− 1.00 (− 3.53, 1.53)	− 0.34 (− 2.03, 2.06)
7–10	− 0.82 (− 5.09, 3.45)	− 0.01 (− 2.23, 2.20)	− 0.08 (− 4.03, 3.87)	− 0.25 (− 2.61, 1.95)	− 1.16 (− 3.41, 1.08)	0.31 (− 1.86, 1.85)
> = 11	1.83 (− 1.56, 5.22)	0.30 (− 1.43, 2.03)	− 1.05 (− 4.25, 2.14)	1.23 (− 0.66, 2.96)	1.00 (− 0.80, 2.80)	1.20^c^ (0.02, 3.04)
Education						
(High school)						
University	− 1.18(− 3.64, 1.29)	0.03 (− 1.43, 1.49)	0.07 (− 2.35, 2.50)	− 0.33 (− 1.67, 1.08)	− 0.75 (− 2.15, 0.64)	− 0.12 (− 1.19, 1.20)
Speaks English at home						
(No)						
Yes	− 2.47 (− 5.04, 0.09)	0.73 (− 0.70, 2.16)	− 0.91(− 3.47, 1.64)	− 0.65 (− 2.19, 0.95)	0.48 (− 0.99, 1.96)	− 0.01 (− 1.66, 0.83)
Weeks of home practice						
(< 2)						
2	− 2.07 (− 6.94, 2.81)	0.05 (− 2.51, 2.61)	4.23 (− 0.63, 9.08)	0.61 (− 1.92, 3.20)	− 0.03 (− 2.60, 2.54)	0.10 (− 2.26, 1.44)
3	− 0.85 (− 5.30, 3.59)	0.05 (− 2.28, 2.38)	3.90 (− 0.59, 8.40)	− 0.68 (− 3.19, 1.86)	− 0.60 (− 3.07, 1.87)	− 0.61 (− 2.61, 0.97)
4	− 0.94 (− 5.40, 3.53)	− 0.56 (− 3.05, 1.94)	4.40 (− 0.16, 8.97)	− 0.03 (− 2.62, 2.62)	− 0.12 (− 2.71, 2.47)	− 0.46 (− 2.51, 1.41)
Group sessions attended						
(< 4)						
4	− 1.76 (− 5.87, 2.35)	− 1.19 (− 3.40, 1.02)	− 2.49 (− 6.53, 1.54)	− 1.21 (− 3.63, 0.84)	0.24 (− 2.16, 2.64)	− 0.33 (− 2.51, 1.41)
5	− 1.69 (− 5.81, 2.42)	− 1.43 (− 3.59, 0.72)	− 2.55 (− 6.49, 1.39)	− 1.96 (− 4.36, 0.09)	0.31 (− 2.07, 2.69)	− 0.72 (− 2.02, 1.40)
Gender						
(Male)						
Female	− 0.78 (− 4.72, 3.15)	0.38 (− 1.72, 2.48)	− 1.11 (− 5.30, 3.09)	− 0.67 (− 3.10, 1.75)	− 0.23 (− 2.42, 1.96)	− 0.80 (− 2.18, 1.25)
Group language						
(Arabic)						
Bangla	1.09 (− 1.49, 3.68)	0.50 (− 1.03, 2.03)	1.97 (− 0.70, 4.64)	1.70^c^ (0.13, 3.22)	1.80^c^ (0.34, 3.26)	2.27^b^ (1.44, 4.01)

The reference category of a variable is in parentheses; a indicates p < 0.001, b indicates p < 0.01, c indicates p < 0.05

Table 4 shows the joint effect of home practice x group attendance and the main effects of age, years in Australia, education, language spoken at home, gender, group language, home practice and group attendance on mental health outcomes, with 95% confidence intervals. The joint effect of home practice x group attendance is not significant for any of the outcomes. On the DASS21, there was a significant decrease in anxiety for participants aged 46–55 and 56–65 compared to those aged below 26 (both p < 0.05). The results for the remaining main effects on outcomes are similar to the ones already reported for Table 3.

**Table 4:** Multiple linear regression of the effects (and their 95% confidence intervals) of age, years in Australia, education, language spoken at home, gender, group language, home practice, group attendance and home practice x group attendance interactions on mental health outcomes (post–pre differences)

Covariates	Difference in K10 score	Difference in K10 Q11 (Days of inability to work)	Difference in K10 Q12 (Days of cutting down work apart from days in Q11)	Difference in DASS depression	Difference in DASS anxiety	Difference in DASS stress
	Coefficient (95% CI)	Coefficient (95% CI)	Coefficient (95% CI)	Coefficient (95% CI)	Coefficient (95% CI)	Coefficient (95% CI)
Age group						
(< 26 years)						
26–35	− 1.35 (− 5.44, 2.75)	− 1.38 (− 3.90, 1.13)	− 2.45 (− 6.90, 1.99)	− 0.41 (− 2.86, 2.03)	− 0.94 (− 3.49, 1.61)	− 0.83 (− 3.29, 1.64)
36–45	− 2.42 (− 7.07, 2.23)	− 1.99 (− 4.74, 0.76)	− 2.79 (− 7.38, 1.81)	− 0.64 (− 3.31, 2.03)	− 2.40 (− 5.06, 0.26)	− 1.28 (− 3.99, 1.44)
46–55	− 2.33 (− 7.11, 2.45)	− 2.25 (− 4.60, 0.46)	− 3.76 (− 8.97, 1.46)	− 1.68 (− 4.44, 1.09)	− 2.85^c^ (− 5.96, 0.27)	− 2.24 (− 5.55, 1.06)
56–65	− 6.23^c^ (− 10.98, − 1.49)	− 3.00^c^ (− 5.68, − 0.32)	− 1.72 (− 6.30, 2.86)	− 1.29 (− 3.97, 1.39)	− 2.64^c^ (− 5.69, 0.41)	− 2.94^c^ (− 5.72, − 0.16)
Years in Australia						
(< 4)						
4–6	− 1.23 (− 5.79, 3.34)	− 0.84 (− 3.39, 1.71)	− 0.44 (− 4.85, 3.96)	0.63 (− 1.99, 3.26)	− 1.00 (− 3.54, 1.55)	− 0.21 (− 2.87, 2.45)
7–10	− 0.69 (− 5.10, 3.71)	− 0.23 (− 2.49, 2.03)	− 0.32 (− 4.34, 3.70)	− 0.11 (− 2.48, 2.25)	− 0.99 (− 3.31, 1.33)	0.49 (− 1.96, 2.93)
> = 11	1.86 (− 1.54, 5.26)	0.19 (− 1.55, 1.93)	− 1.31 (− 4.50,1.87)	1.21 (− 0.62, 3.04)	1.06 (− 0.75, 2.88)	1.19 (− 0.70, 3.07)
Education						
(High school)						
University	− 1.27 (− 3.81, 1.27)	0.09 (− 1.40, 1.58)	− 0.18 (− 2.66, 0.31)	− 0.37 (− 1.78, 1.04)	− 0.91 (− 2.23, 0.64)	− 0.05 (− 1.50, 1.41)
Speaks English at home						
(No)						
Yes	− 2.35 (− 4.97, 0.27)	0.75 (− 0.72, 2.21)	− 0.66 (− 3.29, 1.97)	− 0.64 (− 2.24, 0.96)	0.50 (− 1.00, 2.00)	0.13 (− 1.39, 1.66)
Weeks of home practice						
(< 2)						
2	− 2.62 (− 10.06, 4.83)	− 0.39 (− 4.74, 3.78)	5.07 (− 2.52, 12.65)	0.79 (− 3.06, 4.64)	− 0.28 (− 4.17, 3.61)	− 0.43 (− 4.29, 3.43)
3	− 1.06 (− 9.92, 7.80)	0.33 (− 4.12, 4.77)	7.08 (− 1.90, 16.07)	− 1.81 (− 7.29, 3.66)	− 1.13 (− 6.33, 4.07)	− 0.48 (− 5.49, 4.53)
4	3 .08 (− 9.75, 15.92)	− 2.81 (− 10.06, 4.43)	9.77 (− 3.25, 22.78)	1.67 (− 5.45, 8.79)	1.55 (− 5.68, 8.78)	0.24 (− 7.10, 7.58)
Group sessions attended						
(< 4)						
4	− 2.20 (− 9.79, 5.38)	− 1.64 (− 5.79, 2.52)	0.76 (− 9.55, 8.08)	− 1.30 (− 5.55, 2.95)	− 0.82 (− 5.10, 3.46)	− 1.01 (− 5.27, 3.25)
5	− 1.33 (− 10.06, 7.40)	− 2.05 (− 6.47, 2.37)	− 0.73 (− 9.55, 8.08)	− 2.66 (− 7.27, 1.94)	1.15 (− 3.62, 5.92)	0.17 (− 4.55, 4.89)
Home practice & Group sessions interaction						
(< 2 weeks* < 4 sessions)						
2 wks*4 sessions	1.79 (− 8.06, 11.64)	− 0.25 (− 5.92, 5.43)	− 5.35 (− 15.69, 4.99)	− 0.43 (− 6.01, 5.14)	1.76 (− 3.82, 7.33)	1.01 (− 4.62, 6.65)
2 wks*5 sessions	− 0.04 (− 10.31, 10.22)	2.16 (− 3.62, 7.94)	− 0.42 (− 11.43, 10.60)	0.26 (− 5.54, 6.05)	− 0.91 (− 6.66, 4.83)	− 0.25 (− 6.12, 5.62)
3 wks*4 sessions	0.44 (− 9.93, 10.80)	− 0.07 (− 5.71, 5.58)	− 6.30 (− 16.81, 4.21)	1.42 (− 5.08, 7.92)	1.75 (− 4.54, 8.03)	0.56 (− 5.68, 6.79)
3 wks*5 sessions	0.01 (− 11.00, 11.02)	0.34 (− 5.60, 6.28)	− 4.32 (− 15.82, 7.17)	1.39 (− 5.13, 7.91)	− 0.52 (− 6.87, 5.83)	− 1.50 (− 7.98, 4.98)
4 wks*4 sessions	− 3.74 (− 18.40, 10.91)	3.92 (− 4.38, 12.21)	− 6.82 (− 21.87, 8.22)	− 2.78 (− 11.06, 5.51)	− 1.13 (− 9.36, 7.09)	− 0.50 (− 8.95, 7.94)
4 wks*5 sessions	− 4.57 (− 19.45, 10.30)	2.40 (− 5.84, 10.63)	− 7.15 (− 22.29, 7.99)	− 0.95 (− 9.26, 7.35)	− 2.52 (− 10.88, 5.84)	− 1.76 (− 10.26, 6.74)
Gender						
(Male)						
Female	− 0.77 (− 4.72, 3.17)	0.37 (− 1.73, 2.47)	− 1.19 (− 5.36, 2.97)	− 0.68 (− 3.10, 1.74)	− 0.23 (− 2.42, 1.96)	− 0.88 (− 3.25, 1.49)
Group language						
(Arabic)						
Bangla	1.18 (− 1.44, 3.80)	0.43 (− 1.11, 1.98)	1.96 (− 0.74, 4.65)	1.70^c^ (0.14, 3.26)	1.85^c^ (0.37, 3.33)	2.22^b^ (0.69, 3.75)

The reference category of a variable is in parentheses; a indicates p < 0.001, b indicates p < 0.01, c indicates p < 0.05

### Referrals

Of the Arabic speakers recruited, seven were already connected with a mental health service or professional and 19 new referrals were made. Of the Bangla speakers recruited, four were already seeing a mental health specialist and 11 new referrals were made.

### Participant experiences

Arabic and Bangla speakers who completed the program were equally positive about the experience. Over 90% of participants from both language groups agreed that mindfulness was compatible with their cultural and religious practices, fitted in with their way of life and provided practical strategies to reduce stress (Additional file 3). Further, almost all (92% of Arabic and 99% of Bangla speakers) reported sharing mindfulness skills with others—their immediate and extended family, friends, other community members and colleagues.

### Group sessions

Participants reported that the group sessions were “enjoyable”, “good”, “useful”, “beneficial” and “relevant”. They found the content of all five sessions helpful, including the new concepts and information and the various related exercises. Several participants, especially in the Arabic speaking groups, commented on the cultural and spiritual relevance and group discussion (“sharing”, “connection” and “meeting others”). Overall, there was little that was considered unhelpful. The material was “clearly presented” and “understandable” and the facilitators engaged the group well. When present, as was the case in two groups, children were a distraction, as were interruptions and background noise. Most participants wanted more information and more sessions, or an opportunity to take part in future programs.

### Home practice

Participant feedback on home practice across the five weeks was particularly instructive. After the first group session, participants were highly motivated (“will try to practice”). As they practiced the mindfulness exercises and experienced the benefits (e.g. feeling more relaxed, reduced stress and improved sleep), they grew in confidence and increasingly applied the new skills in everyday life (e.g. during prayers, reading the Quran, interacting with family, cooking, doing chores and before going to bed) and shared them with others. By the third week, many participants were practicing with family or a friend. Both Arabic and Bangla speakers reported improved family relationships as well as improvements in their own wellbeing. Some noted that it was difficult to find the opportunity to practice or to carry out the exercises (e.g. finding it hard to focus or relax, or struggling with pain). Despite the challenges, they continued trying to practice (“needs to be a habit”).

Content analysis of all the qualitative data, including both written and verbal feedback, highlighted the main benefits experienced by participants: skills development and personal growth, increased ability to cope with ongoing stressors, and improved wellbeing and relationships. The quotations in Box 2 are illustrative.

Box 2. Participant-reported benefits ***Skills development and personal growth***

“I’m happy that I’ve done the mindfulness program as I learnt about myself. I enjoyed the exercises and I practice the breathing exercise daily. It has made me more thoughtful, compassionate and wise.” (Bangla speaker).

“I wish I had attended all sessions. Having sick children and no one to look after them limited my attendance. I am more mindful when I talk to my children. I am more mindful in my prayers. Trying to practice the skills whenever I can.” (Arabic speaker).

“The program restored my confidence in myself and others. My outlook on the world has improved so much. I have more acceptance of things that are out of my control. My relationship with myself is much better and I want to do a lot of things for myself.” (Arabic speaker).

***Ability to cope with ongoing stressors***

“It’s great information. Very important to know because there is a way to deal with stress and taking care of myself.” (Arabic speaker).

“Mindfulness skills have helped me calm down during stressful situations with my family. I have also shared these skills with my mum.” (Arabic speaker).

“This course taught me to manage my tough situation in life while experiencing a health condition. The skills I learnt will help me for a lifetime.” (Bangla speaker).

“The exercises helped me reduce stress related to assignments and schoolwork.” (Arabic speaker).

***Wellbeing and relationships***

“I practice mindfulness in the evening and they help me sleep well at night.” (Bangla speaker).

“Being aware in my prayer allowed me to have a deeper and more meaningful connection with God.” (Arabic speaker).

“I used to think a lot about my past but now I think about the present.” (Arabic speaker).

“I did the breathing exercise with my 6-year-old. It is helping to promote positive behaviour and time management for both of us.” (Bangla speaker).

“The program has taught me to cope with negativity I hear from people and since attending the program I have started feeling good about myself and now I go outside to meet people.” (Bangla speaker).

## Discussion

Despite a growing evidence base and wide-spread acceptance among the general population, there has been limited mindfulness-based research in ethnic minorities in western immigrant countries [22, 23]. Phase 3 of this ongoing research, with its successful community engagement, high retention rate and positive outcomes, provides further support for the cultural acceptability and clinical utility of mindfulness among Arabic-speaking community members in Sydney and has demonstrated its value for Bangla speakers.

### Engagement and delivery

The widespread underutilisation of mental health services by people from CALD communities in Australia is well-documented [4]. Barriers include cultural and religious beliefs, low English proficiency and lack of knowledge and experience with the health system, together with the stigma and shame attached to mental illness [24–26]. Working with CALD community organisations and groups, 271 people, mostly married women, were recruited to 23 mindfulness group programs. The Arabic speakers included both Muslims and Christians and one-fifth were Australian born. Nearly all the Bangla speakers were Muslims and all were born overseas.

The program was delivered by bilingual co-facilitators (a mental health professional and a community worker, both female) at various community venues. Program materials were translated into Arabic and Bangla, and all groups were facilitated in the community language, sometimes with supplementary English. Importantly, the experienced facilitators were able to respond to the needs of different groups without compromising its fidelity or effectiveness. Concepts were explained and exercises were introduced in accordance with group members’ backgrounds and understanding.

Participant retention was high despite the time commitment involved (2.5 h a week for five weeks), as was engagement in home practice. Active participation, including completion of homework assignments or practice between sessions, enhances cognitive and behavioural therapy outcomes [27]. Time spent in formal home practice has been shown to be significantly related to the extent of improvement in measures of mindfulness and psychological wellbeing [28].

### Cultural acceptability

Cultural acceptability was evident in the high retention rates for both language groups, and in post-program responses to the mindfulness knowledge and attitudes questionnaire. It was also reflected in the weekly feedback forms and comments to the group facilitators, and the extensive sharing with family, friends and other community members.

### Clinical utility

In both language groups, levels of psychological distress, depression, anxiety and stress were reduced after four weeks with fewer people in the ‘severe’ and ‘extremely severe’ categories. Median differences on the psychometric measures pre and post-program were statistically significant; replicating findings from Phases 1 and 2 of this research [9–11]. Participants also showed significant improvement in daily functioning. Phase 1, which included a 12-week follow-up, found that all but four of the 70 Arabic-speaking participants continued with mindfulness practice beyond the 5-week group program and that clinical improvement was maintained [9]. One advantage of the group format is the opportunity for meeting others with similar interests. It is possible that such connections, as well as sharing mindfulness skills with family and friends, may contribute to the positive outcomes being sustained through ongoing peer support, however we have no evidence for this. Importantly, the program provided a soft-entry point into the healthcare system for those requiring additional care, with 30 new referrals to mental health specialists.

Analysis of the effects of sociodemographic variables (age, years in Australia, education, language spoken at home, gender and group language) and intervention exposure (home practice and group attendance) on mental health outcomes produced few significant results. Regressions including main effects only and main effects plus the joint effect of home practice x group attendance produced similar findings. Compared to participants under 26 years, those aged 56–65 showed significantly greater improvement on the K10 + (psychological distress and days of inability to work due to mental health issues) and those aged 46–55 and 56–65 showed significantly greater improvement on the DASS21 stress subscale. When the joint effect of home practice x group attendance was factored in, those aged 46–55 and 56–65 also showed significantly greater improvement on the anxiety subscale. It is not obvious why this should be so; possibly the older women have reached a life stage where they are more ready and able to focus on their mental health and wellbeing. Nevertheless, these results suggest additional attention should be paid to targeting the younger population.

Years living in Australia (possibly an indicator of acculturation) was significantly associated with a reduction in stress in the main effects model. Attending a Bangla-speaking group, rather than an Arabic-speaking group, was significantly associated with an increase in anxiety, depression and stress. Again, the reason is not obvious. The Bangla speakers were generally younger, better educated and had spent fewer years in Australia. As a new and emerging community, the Bangla community has a much smaller population, significant settlement issues and less community infrastructure than the Arabic community [8].

Qualitative data confirmed the program’s clinical utility, with participants reporting that they learned a lot and that mindfulness practice enhanced daily functioning and contributed to improved family relationships. Participants most commonly reported applying mindfulness in family interactions and prayer, activities of high importance within the Arabic and Bangla-speaking communities. Group discussion was seen as a positive feature. Encouraged by the group facilitators and early benefits, they took their home practice seriously and shared the new skills with their social networks. Although we did not measure this, sharing the program information and materials and practicing mindfulness with others undoubtedly increased community mental health literacy, i.e. “knowledge about mental disorders that are associated with their recognition, management and prevention” [29]. These findings are consistent with other studies demonstrating the benefits of culturally-tailored mental health promotion and mental health literacy training on the wellbeing and mental health literacy of members of the Arabic-speaking community in Sydney [30, 31].

### Capacity building

The broader project in which this study was nestled included two other components designed to extend the program’s reach and improve access to evidence-based mental health care for CALD community members: development of in-language audio resources and a facilitator training program. Audio resources in Arabic, Bangla and English were made available on USB to past and future participants in order to assist with their ongoing mindfulness practice. Since December 2019, they have also been available on the NSW Multicultural Health Communication Service website [32]. The facilitator training program was designed to build workforce capacity across primary and specialist mental health care settings. Workshop content covered core mindfulness concepts and techniques and how cultural and religious tailoring of interventions enhances acceptability to CALD communities. Over 70 bilingual mental health clinicians and bilingual community workers now have the skills to facilitate mindfulness groups to their respective communities.

The demonstrated clinical utility of the group mindfulness program with Arabic and Bangla speakers, and the cultural acceptability of the program materials, resulted in further funding to continue roll-out to these community groups. In March 2020, in response to the novel coronavirus, COVID-19, Australia closed its international borders, introduced social distancing and placed limits on the number of people who could gather. As health services were not able to offer face-to-face groups, the clinical lead adapted program resources to support the Arabic-speaking community through regular text messages via established community networks. These messages encouraged mindfulness practice at a time of changing and challenging circumstances. The clinical lead also recorded a 5-min video (in Arabic and English) to introduce mindfulness concepts and link people with the audio resources, which was disseminated through social media and community networks. Subsequently, the mindfulness program was adapted for delivery via videoconferencing and offered as a one-off session to previous participants or as a shortened multi-session program for new and returning participants. A similar virtual group program has been implemented with the Bangla-speaking community. Accelerated by COVID19, there is emerging international research on the use and effectiveness of mindfulness interventions delivered via telemedicine and online [33–35].

### Strengths and limitations

As an effectiveness study [13], bringing together government services and non-government and community organisations, Phase 3 demonstrates how the group mindfulness program is able to deliver outcomes under normal operational conditions. Both the K10 and the DASS21 are self-report measures; nevertheless, they have very good psychometric properties and translated versions have been used with a broad range of study populations, including Arabic and Bangla speakers [9, 19, 20, 31]. We did not assess mindfulness using a validated questionnaire, such as the Mindfulness Attention Awareness Scale or the Five Facet Mindfulness Questionnaire [36, 37]. This quasi-experimental study [38] did not include a control/comparison group, nor did we do any formal follow-up. We were unable to collect post-program data from people who dropped out. Given the high scores on the mental health measures at entry, some regression towards the mean is to be expected. In the absence of a control group it is impossible to conclusively demonstrate cause and effect, however the qualitative data suggests that the intervention did have an impact. Intention to treat analysis accounts for dropouts and incomplete program adherence. Most of the group mindfulness programs were targeted at women, and all were facilitated by women. Over 90% of participants were aged over 26 years (and married women with children). Further research is needed to extend the intervention to men and young people, as well as other migrant communities.

## Conclusions

Translation and cultural adaptation of evidence-based interventions which are then delivered in community settings enables engagement with CALD community members who would not otherwise access mental health services, as well as high retention rates and positive mental health outcomes. Across multiple and diverse groups of Arabic and Bangla speakers in Sydney, the community-based group mindfulness program was shown to have high levels of cultural acceptability and relevance. It resulted in clinically and statistically significant improvements in mental health outcomes, facilitated access to mental health care and boosted mental health literacy. This innovative, low-intensity, in-language mental health intervention that was originally developed for Arabic speakers is scalable and transferable—with cultural tailoring—to Bangla speakers. Future directions include further experimentation with technology-mediated mindfulness interventions.

## Supplementary Information

**Additional file 1. **Sociodemographic characteristics by language group – Participants recruited.

**Additional file 2.** Pre and post-program K10 and DASS21 categories by language group – Participants retained.

**Additional file 3.** Pre and post-program mindfulness knowledge and attitudes by language group – Participants retained.

## Data Availability

The data sets are not publicly available as they contain information that could potentially re-identify individuals, but are available from LW (author) upon reasonable request and with relevant ethical approval. Program materials are available from HS (author).

## Abbreviations

CALD: Culturally and Linguistically Diverse
CESPHN: Central and Eastern Sydney Primary Health Network
DASS21: Depression Anxiety and Stress Scale – 2i items
K10: Kessler Psychological Distress Scale
NSW: New South Wales
SESLHD: South Eastern Sydney Local Health District
SLHD: Sydney Local Health District
