# Supplementary material for: Effectiveness of a Community-based Group Mindfulness Program tailored for Arabic and Bangla-speaking Migrants
Source: Int J Ment Health Syst. 2021 Apr 13;15:32. doi: 10.1186/s13033-021-00456-0 (PMC8042358; doi:10.1186/s13033-021-00456-0)
Supplement: Supplementary file 1 — Additional file 1. Sociodemographic characteristics by language group – Participants recruited. [file 13033_2021_456_MOESM1_ESM.pdf]

Additional file 1
Sociodemographic characteristics by language group – Participants recruited

Variable	Arabic speakers (N=168)		Bangla speakers (N=103)	
	n	%	n	%
**Age group (years)**				
16-25	24	14.3	7	6.8
26-35	41	24.4	45	43.7
36-45	28	16.7	35	34.0
46-55	25	14.9	9	8.7
56-65	50	29.8	7	6.8
**Gender**				
Male	28	16.7	6	5.8
Female	140	83.3	97	94.2
**Country of birth**				
Australia	35	20.8		
Lebanon	49	29.2		
Egypt	31	18.5		
Iraq	20	11.9		
Syria	15	8.9		
Sudan	4	2.4		
Jordan	4	2.4		
Bangladesh			101	98.1
India			1	1.0
Ecuador			1	1.0
Other*	8	4.8		
**Years in Australia**				
0-3	33	19.6	27	26.2
4-6	9	5.4	19	18.4
7-10	15	8.9	23	22.3
11+	105	62.5	33	32.0
**Language spoken at home** *(n=167, missing data for 1)*				
Arabic	70	41.9		
Arabic + English ± Other	81	48.5		
Arabic + Assyrian	6	3.6		
Assyrian	3	1.8		
Kurdish	1	0.6		
English	6	3.6		
Bangla			76	73.8
Bangla + English			26	25.2
English + Spanish			1	1.0
**Religion**				
Muslim	105	62.5	95	92.2
Christian	62	36.9	3	2.9
Buddhist			2	1.9

Hindu			2	1.9
Other			1	1.0
None	1	0.6		
**Education** *(n=165, missing data for 3)*				
Year 12 or less	76	45.2	29	28.2
Trade certificate	24	14.3	14	13.6
University	65	38.7	60	58.3
**Marital status** *(n=167, missing data for 3)*				
Never married	28	16.7	1	1.0
Married/De facto	113	67.3	96	93.2
Divorced/Separated	16	9.5	5	4.9
Widowed	9	5.4	1	1.0
**Number of children**				
0	39	23.2	7	6.8
1+	128	76.2	96	93.2

*Dominican Republic, Iran, Kuwait, Morocco, Palestine, Saudi Arabia, Senegal and Tunisia
