# Supplementary material for: Effectiveness of a Community-based Group Mindfulness Program tailored for Arabic and Bangla-speaking Migrants
Source: Int J Ment Health Syst. 2021 Apr 13;15:32. doi: 10.1186/s13033-021-00456-0 (PMC8042358; doi:10.1186/s13033-021-00456-0)
Supplement: Supplementary file 2 — Additional file 2. Pre and post-program K10 and DASS21 categories by language group – Participants retained. [file 13033_2021_456_MOESM2_ESM.pdf]

**Additional file 2**
**Pre and post-program K10 and DASS21 categories by language group – Participants retained**

| Measure | Arabic speakers (N=131) | | | | Bangla speakers (N=87) | | | |
|---|---|---|---|---|---|---|---|---|
| | Pre-program | | Post-program | | Pre-program | | Post-program | |
| | n | % | n | % | n | % | n | % |
| **DASS21 depression** | | | | | | | | |
| Normal (0-4) | 33 | 25.2 | 80 | 61.1 | 27 | 31.0 | 50 | 57.5 |
| Mild (5-6) | 8 | 6.1 | 17 | 13.0 | 11 | 12.6 | 17 | 19.5 |
| Moderate (7-10) | 36 | 27.5 | 24 | 18.3 | 21 | 24.1 | 10 | 11.5 |
| Severe (11-13) | 21 | 16.0 | 2 | 1.5 | 13 | 14.9 | 6 | 6.9 |
| Extremely Severe (14+) | 33 | 25.2 | 8 | 6.1 | 15 | 17.2 | 4 | 4.6 |
| **DASS21 anxiety** | | | | | | | | |
| Normal (0-3) | 33 | 25.2 | 82 | 62.6 | 30 | 34.5 | 48 | 55.2 |
| Mild (4-5) | 17 | 13.0 | 20 | 15.3 | 12 | 13.8 | 14 | 16.1 |
| Moderate (6-7) | 15 | 11.5 | 12 | 9.2 | 7 | 8.0 | 6 | 6.9 |
| Severe (8-9) | 12 | 9.2 | 5 | 3.8 | 6 | 6.9 | 12 | 13.8 |
| Extremely Severe (10+) | 54 | 41.2 | 12 | 9.2 | 32 | 36.8 | 7 | 8.0 |
| **DASS21 stress** | | | | | | | | |
| Normal (0-7) | 34 | 26.0 | 96 | 73.3 | 39 | 44.8 | 67 | 77.0 |
| Mild (8-9) | 17 | 13.0 | 18 | 13.7 | 8 | 9.2 | 8 | 9.2 |
| Moderate (10-12) | 26 | 19.8 | 7 | 5.3 | 15 | 17.2 | 7 | 8.0 |
| Severe (13-16) | 28 | 21.4 | 9 | 6.9 | 12 | 13.8 | 2 | 2.3 |
| Extremely Severe (17+) | 26 | 19.8 | 1 | 0.8 | 13 | 14.9 | 3 | 3.4 |
| **K10** | | | | | | | | |
| Well (10-19) | 29 | 22.1 | 86 | 65.6 | 28 | 32.5 | 63 | 72.4 |
| Mild (20-24) | 26 | 19.8 | 22 | 16.8 | 22 | 25.3 | 13 | 14.9 |
| Moderate (25-29) | 26 | 19.8 | 15 | 11.5 | 15 | 17.2 | 7 | 8.0 |
| Severe (30-50) | 50 | 38.2 | 8 | 6.1 | 22 | 25.3 | 4 | 4.6 |
