# Supplementary material for: Effectiveness of a Community-based Group Mindfulness Program tailored for Arabic and Bangla-speaking Migrants
Source: Int J Ment Health Syst. 2021 Apr 13;15:32. doi: 10.1186/s13033-021-00456-0 (PMC8042358; doi:10.1186/s13033-021-00456-0)
Supplement: Supplementary file 3 — Additional file 3. Pre and post-program mindfulness knowledge and attitudes by language group – Participants retained. [file 13033_2021_456_MOESM3_ESM.pdf]

Additional file 3
Pre and post-program mindfulness knowledge and attitudes by language group – Participants retained

Statement	Arabic speakers (N=131)				Bangla speakers (N=87)			
	Pre-program		Post-program		Pre-program		Post-program	
	n	%	n	%	n	%	n	%
**1. Mindfulness is a form of meditation**								
Strongly Agree	15	11.5	34	26.0	4	4.6	39	44.8
Agree	34	26.0	76	58.0	18	38.3	41	47.1
Neither agree nor disagree	4	3.1	2	1.5	2	2.3	1	1.1
Disagree	1	0.8	9	6.8	2	2.3	2	2.3
Strongly Disagree	1	0.8	9	6.8	1	1.1	4	5.1
Don't know	76	58.0	1	0.7	60	69.0	0	0
**2. Mindfulness is about focussing on the past and the future**								
Strongly Agree	8	6.1	11	8.4	6	6.9	2	2.3
Agree	25	19.1	8	6.1	14	16.1	3	3.4
Neither agree nor disagree	5	3.8	0	0	1	1.1	1	1.1
Disagree	13	9.9	39	29.8	4	4.6	29	33.3
Strongly Disagree	9	6.9	72	55.0	3	3.4	52	59.8
Don't know	71	54.2	1	0.8	59	67.8	0	0
**3. Mindfulness uses the breath to improve concentration**								
Strongly Agree	13	9.9	63	48.1	6	6.9	36	41.4
Agree	44	33.6	57	43.5	20	23.0	49	56.3
Neither agree nor disagree	4	3.1	4	3.1	1	1.1	1	1.1
Disagree	0	0	2	1.5	1	1.1	1	1.1
Strongly Disagree	0	0	2	1.5	1	1.1	0	0
Don't know	70	53.4	3	2.3	58	66.6	0	0
**4. Mindfulness is a type of physical activity**								
Strongly Agree	5	3.8	17	13.0	3	3.5	5	5.7
Agree	20	15.3	42	32.1	12	13.8	21	24.1
Neither agree or disagree	14	10.7	18	13.7	3	3.5	19	21.8
Disagree	10	7.6	36	27.5	4	4.6	33	37.9
Strongly disagree	3	2.3	15	11.5	2	2.3	7	8.0
Don't know	79	60.3	3	2.3	64	73.6	2	2.3
**5. Mindfulness is compatible with existing cultural and religious practices**								
Strongly Agree	16	12.2	67	51.1	8	9.2	47	54.0
Agree	29	22.1	58	44.3	12	13.8	35	40.2
Neither agree nor disagree	5	3.8	3	2.3	0	0	0	0
Disagree	2	1.5	2	1.5	3	3.5	3	3.5
Strongly disagree	2	1.5	1	0.8	2	2.3	2	2.3
Don't know	77	58.8	0	0	62	71.3	0	0
**6. I would be willing to learn new ways of improving my wellbeing through Mindfulness**								
Strongly Agree	85	64.9	98	74.8	38	43.7	56	64.4
Agree	40	30.5	31	23.7	42	48.3	30	34.5
Neither agree nor disagree	1	0.8	1	0.8	0	0	0	0
Disagree	0	0	0	0	0	0	0	0

Strongly Disagree	2	1.5	0	0	2	2.4	1	1.1
Don’t know	3	2.3	1	0.8	5	5.7	0	0
**7. Mindfulness fits in with my way of life**								
Strongly Agree	24	18.3	78	59.5	7	8.0	50	57.5
Agree	36	27.5	50	38.2	14	16.1	37	42.5
Neither agree nor disagree	4	3.1	2	1.5	0	0	0	0
Disagree	1	0.8	0	0	0	0	0	0
Strongly disagree	1	0.8	0	0	2	2.3	0	0
Don’t know	65	49.6	1	0.8	64	73.6	0	0
**8. Mindfulness offers practical strategies to reduce stress**								
Strongly Agree	30	22.9	95	72.5	9	10.3	68	78.2
Agree	42	32.1	35	26.7	15	17.2	18	20.7
Neither agree nor disagree	1	0.8	1	0.8	0	0	0	0
Disagree	0	0	0	0	0	0	0	0
Strongly Disagree	0	0	0	0	2	2.3	1	1.1
Don’t know	58	44.3	0	0	61	70.1	0	0
